# EXPLORING EXERCISE INTOLERANCE IN ADULT PATIENTS WITH PERSISTENT POST-CONCUSSION SYMPTOMS AFTER MILD TRAUMATIC BRAIN INJURY

**DOI:** 10.2340/jrm.v57.43931

**Published:** 2025-11-05

**Authors:** Lars-Johan V. VALAAS, Helene L. SOBERG, Mari S. RASMUSSEN, Sophie E. STEENSTRUP, Ingerid KLEFFELGÅRD

**Affiliations:** 1 Center for Habilitation and Rehabilitation Models and Services (CHARM), Faculty of Medicine, Institute of Health and Society, University of Oslo, Oslo; 2 Department of Physical Medicine and Rehabilitation, Oslo University Hospital, Oslo; 3 Department of Rehabilitation Science and Health Technology, Faculty of Health Sciences, Oslo Metropolitan University, Oslo, Norway

**Keywords:** brain concussion, mild traumatic brain injury, brain injuries, traumatic, exercise test, post-concussion syndrome, post-traumatic headache

## Abstract

**Objectives:**

Explore exercise intolerance with the Buffalo Concussion Treadmill Test (BCTT) in adult patients with persisting post-concussion symptoms (PCS). Examine the association between exercise intolerance and persistent post-concussion symptom burden. Explore factors associated with the BCTT symptom threshold.

**Design:**

Cross-sectional study.

**Patients:**

100 patients (mean age 36.9 [SD 10.6] years; 58% women) with mild traumatic brain injury and persistent PCS.

**Methods:**

Exercise intolerance was assessed using descriptive statistics and group comparisons. Regression models were applied testing the association between exercise intolerance and symptom burden, and to explore factors associated with symptom threshold.

**Results:**

81% tested positive for exercise intolerance. Patients with exercise intolerance were younger, had a shorter time since injury, more symptoms, and poorer quality of life. Higher symptom burden was associated with increased odds for exercise intolerance (OR: 1.07, 95%CI: 1.01 to 1.13, ***p*** = 0.023). Higher heart rate at start of BCTT was associated with a higher symptom threshold (***B***: 0.34, 95%CI: 0.17 to –0.50, ***p*** < 0.001), while greater anxiety symptoms were associated with lower symptom threshold (***B***: –0.86, 95%CI: –1.6 to –0.12, ***p*** < 0.023) with R^2^ = 0.21.

**Conclusion:**

Exercise intolerance was confirmed in over 80% of the patients. Assessments of exercise intolerance might contribute to a broader understanding and improved rehabilitation of these patients.

## LAY ABSTRACT

Many people experience persisting symptoms after mild traumatic brain injury (mTBI). These symptoms can worsen during exercise. In this study, we included 100 adult patients with persistent post-concussion symptoms, 3 to 24 months after injury, and tested them for exercise intolerance with a standardized treadmill test. Overall, 81% of the patients had exercise intolerance. Greater symptom burden was associated with higher likelihood of exercise intolerance. More research is needed for a better understanding of important factors that can explain exercise intolerance. Our study found that in patients with exercise intolerance, a higher pre-test heart rate was associated with higher symptom threshold, whereas more anxiety was associated with lower symptom threshold. These findings show that exercise intolerance is common in people with persisting post-concussion symptoms and is linked to both physical and psychological factors. Testing for exercise intolerance may help tailor treatment and improve recovery for these patients.

Exercise intolerance (EI) after mild TBI (mTBI) (1) remains poorly understood in adults with persistent post-concussion symptoms (PCS) (2–5). EI is defined as the inability to exercise to near-age maximal heart rate (HRmax) due to exacerbation of PCS, such as headache, dizziness, or nausea (1). The exact prevalence of EI in patients with persisting PCS is not known (1–3). PCS are reported in between 35% and 46% of patients 3 months after mTBI and may persist up to6 months (6), and beyond (7).

The transitioning from passive management of PCS with rest until symptoms resolve, to active management where patients are advised to be active within symptom limits, has shown promising results in sports-related PCS in adolescents (8, 9). The Buffalo Concussion Treadmill Test (BCTT) (10), a symptom-guided incremental test designed to evaluate EI, has gained much attention in the last years (3, 7, 11–13). The BCTT guides a gradual return to physical activity based on a symptom threshold, defined as the HR associated with exacerbation of PCS (1).

Evidence is limited on which factors are involved in EI after mTBI; however, autonomic dysfunction is suggested as an underlying mechanism (13, 14). Autonomic dysfunction is hypothesized to lead to reduced ability to adjust and maintain cerebral blood flow, blood pressure, and HR in response to different levels of physical activity (15). General deconditioning and post-injury psychological distress, secondary physical inactivity due to symptom exacerbation, and recommendations to rest provided by healthcare practitioners are hypothesized to contribute to EI and maintenance of the PCS (15–17).

There is a paucity of literature exploring EI in adult patients with persistent PCS (7, 14, 15). Exploring the relationship between performance-based measures like the BCTT and PCS may contribute to our understanding of EI. EI has primarily been studied in adolescents, and sports-related mTBI populations. However, the majority of mTBI patients referred to trauma centres have greater variation in age, injury mechanisms, life settings, and physical activity levels. In addition, few studies have examined EI in the persistent phase after injury, which is the phase when mTBI patients often are referred to specialized health services.

To improve the understanding of EI in adult patients with persisting PCS and self-reported exacerbation of symptoms during exercise or physical activity we aimed to (*i*) describe and explore EI assessed with the BCTT; (*ii*) explore the association between BCTT results (EI/ET) and persistent PCS burden: and (*iii*) explore factors associated with the BCTT symptom threshold in the patients with EI.

We hypothesized that: (*i*) EI is confirmed with the BCTT in most patients with self-reported exacerbation of symptoms during exercise or physical activity, (*ii*) a positive BCTT for exercise intolerance is associated with higher PCS burden, and (*iii*) EI is associated with a higher PCS burden, more psychological distress, higher fatigue levels, and lower levels of physical activity.

## METHODS

### Study design

This was a cross-sectional study exploring baseline data in a randomized controlled trial investigating the effect of sub-symptom threshold aerobic exercise in patients with persisting PCS and EI (clinicaltrial.gov #NCT05086419). The Regional Committee for Medical and Health Research Ethics South-East Norway (#256109) approved the study.

The study was conducted at the TBI outpatient clinic at the Department of Physical Medicine and Rehabilitation, Oslo University Hospital, the regional trauma referral centre for South-East Norway (18). Patients were referred to the outpatient TBI clinic from the Emergency Department, Neurosurgical Department, and general practitioners in the greater Oslo area. Potentially eligible patients for this study were screened by physicians at the outpatient clinic, informed about the study, and recruited consecutively from March 2022 to November 2023. Written informed consent was obtained from all patients. The study adheres to the STROBE guideline for cross-sectional studies.

### Participants and procedures

Patients between 18 and 60 years, diagnosed with an mTBI (5), who had persistent PCS (3 to 24 months post-injury), and who reported exacerbation of symptoms during physical activity or exercise were invited to the study. Patients were excluded if they had severe neurological or psychiatric conditions documented in their medical records, cardiovascular disease and extremity injuries that contraindicated exercise testing, ongoing substance abuse, and insufficient proficiency in Norwegian.

The baseline assessment took approximately 90 min where the patients filled out patient-reported outcome measures (PROMs) and the researcher (author LJVV) registered demographics, injury-related characteristics (injury severity, mechanisms, and imaging findings), and comorbidities prior to the BCTT procedure.

### Demographics and injury-related characteristics

Demographic variables included age, sex, marital status, education, employment status and sick leave, and body mass index, blood pressure (19), and self-reported comorbidities.

The following injury characteristics were extracted from the medical records: time since injury at baseline, Glasgow Coma Scale score (GCS, range: 13–15), post traumatic amnesia (PTA), loss of consciousness (LOC), performed MRI/CT, and complicated (positive neuroimaging findings) or uncomplicated (negative neuroimaging findings) (6, 20). Injury mechanism was categorized as fall, traffic and bike accidents, sports-related injuries, and other.

### Buffalo Concussion Treadmill

BCTT is an incremental treadmill test (9) where patients walk on a treadmill at a brisk pace (5.2–5.8 km/h); with a 1% incline increase each minute until cessation. Prior to starting the test, and each minute during the test, the test administrator records the patient’s PCS-related symptoms, symptom intensity on a 0–10 numeric rating scale (NRS 0–1, 0 = no symptom to 10 = worst imaginable symptom), perceived rating of physical exertion on the Borg RPE scale 6–20 (21), and HR (polar H10). The test is terminated by the following stop criteria: (*i*) exercise intolerant (EI): exacerbation of PCS reported as ≥ 3 points on the 0–10 NRS (a point more for a new symptom), (*ii*) exercise tolerant (ET): reaching 90% of estimated max HR (211–0.64*age) or ≥ 18 Borg RPE without symptom exacerbation.

### Outcomes

Several outcomes arose from BCTT. We recorded positive and negative tests for EI, using a cut-off of ≤ 90% age-estimated HRmax as a positive test. For the patients with EI, the symptom threshold was the HR at which patients reported a symptom exacerbation, defined as increase of ≥ 3 points on the NRS 0–10, with a new symptom counted as +1 point (1). We adjusted the symptom threshold using percentage reached on the BCTT by age-estimated HRmax (22). Additionally, other BCTT outcomes presented in this paper included Borg RPE, symptom rating (NRS 0–10), and HR that was measured each minute, and time-to-stop (in minutes). We denote first HR observation prior to start, standing at the treadmill as BCTT HR_0._

### Patient-reported outcome measures

RPQ is a TBI-specific PROM widely applied in the mTBI population. It comprises 16-items assessing physical, cognitive, and emotional symptoms over the past week compared with pre-TBI (23). Items are rated on a 0–4 ordinal scale, from 0 (no problem) to 4 (severe problem), resulting in a sum score of 0–64, where higher scores indicate worse symptoms, and a higher symptom burden.

Health-related quality of life (HRQOL), psychological and physical functioning were assessed using the following validated questionnaires: Quality of Life After Brain Injury – Overall Scale (QOLIBRI-OS) measured HRQOL (range: 0–100, worst–best) (24). Depressive symptoms were assessed with the Patient Health Questionnaire (PHQ-9, range: 0–27, best–worst, clinical cut-offs, mild: 5–9, moderate: 10–14, severe: ≥ 15 (25). Anxiety symptoms were measured by the Generalized Anxiety Disorder scale (GAD-7, range: 0–21, best–worst, clinical cut-offs, mild: 5–9, moderate: 10–15, severe: ≥ 15) (26). Fatigue severity was assessed using the Fatigue Severity Scale (FSS-9, range: 1–7, best–worst, with high fatigue ≥ 5 points) (27). Physical activity was evaluated using International Physical Activity Questionnaire (IPAQ), which estimates the total metabolic equivalent task minutes per week (MET-hour), and hours spent sitting (28).

### Sample size

The sample size was calculated for the ongoing RCT (29) and not specifically for the analyses performed in this cross-sectional study.

### Missing data

The medical doctor (NA) estimated missing GCS scores based on the medical record.

### Statistical methods

Descriptive data are presented as means and standard deviations (SD) for normally distributed continuous variables and as medians with 25th and 75th percentile (p25, p75) for non-normally distributed data, with between-groups (EI/ET) analyses conducted accordingly. *T*-tests were used to compare continuous data, and categorical data were analysed using the χ^2^ test or Fisher’s exact test. Symptom threshold and minutes-to-stop on the BCTT are illustrated with survival plots, with log-rank tests for between-group comparison.

We used logistic regression to assess the association between the BCTT result (EI or ET) as the dependent variable, and RPQ sum scores, representing the PCS burden, as the independent variable (*n* = 100). The association was adjusted for age and sex, reporting odds ratios (OR).

We used multiple linear regression to analyse factors associated with EI using the BCTT symptom threshold (%HRmax) as the dependent variable (*n* = 81). We included the following independent variables based on the literature, clinical expertise, and pre-specified hypothesis: sex (female/male), months since injury, BCTT HR_0_ (15), RPQ sum score, depression (PHQ-9), anxiety (GAD-7), fatigue (FSS-9), and physical activity level (IPAQ MET-hour). We report coefficients (unstandardized betas), 95% CIs, *p*-values, variance inflation factor scores (VIF), and goodness-of-fit. An alpha level of 5% was set.

Analyses were performed in RStudio (v.2024.09.01; R Foundation for Statistical Computing, Vienna, Austria) using base R and the survival package (30) for time-to-stop data.

## RESULTS

A total of 103 patients were recruited to this study. Three patients were excluded due to aberrant HR, extremity injury, and post COVID-19 condition. The patients were included in the study at a mean 7 (SD: 3.9) months post-injury. Mean age was 36.9 years (SD: 10.6), the majority (58%) were female, and 95% were employed or students. For detailed patient demographics and injury characteristics, see Table I. There were no missing data from the PROMS or BCTT.

**Table I:** Participants, demographic and injury characteristics

Item	Overall *n* = 100	Exercise intolerant *n* = 81	Exercise tolerant *n* = 19	*p*-value
Demographic and personal factors				
Age, mean (SD)	36.9 (10.6)	35.8 (10.6)	41.9 (9.5)	***p* = 0.022**
Sex, *n* (%)				
Female	58 (58)	47 (58)	11 (58)	*p* = 0.992
Male	42 (42)	34 (42)	8 (42)	
Marital status, *n* (%)				
Single	46 (46)	40 (50)	6 (68)	*p* = 0.161
Partnered	54 (54)	41 (50)	13 (32)	
Education, > 14 years, *n* (%)				
No	13 (13)	11 (13.6)	2 (10.5)	*p*>0.999
Yes	87 (87)	70 (86.4)	17 (89.5)	
Work status, *n* (%)				
Employed,	95 (95)	78 (78)	17 (89)	*p* = 0.558
Not employed	4 (4)	3 (3)	1 (5)	
Sick leave, *n* (%)				
– < 30%	13 (13)	10 (13)	3 (16)	*p* = 0.191
– 30–69.9%	23 (23)	17 (21)	6 (32)	
– > 70%	63 (63)	53 (66)	10 (53)	
BMI, mean (SD)	24.4 (3.3)	24.4 (3.2)	24.4 (3.6)	*p* = 0.974
Blood pressure, *n* (%)				
Normal	42 (42)	38 (47)	4 (21)	***p* = 0.045**
High normal	35 (35)	28 (34.5)	7 (37)	
Hypertension (grade 1–3)	23 (23)	15 (18.5)	8 (42)	
Comorbidity present, *n* (%)				
0	41 (41)	30 (37)	11 (58)	*p* = 0.221
1	39 (39)	33 (41)	6 (32)	
2 or more	20 (20)	18 (22)	2 (10)	
Comorbidities, *n* (%)				
None	40 (40)	30 (37)	10 (53)	*p* = 0.323
Headaches	21 (21)	19 (20)	2 (11)	*p* = 0.348
Musculoskeletal	31 (31)	28 (34)	3 (16)	*p* = 0.188
Cardiovascular and respiration	10 (10)	8 (10)	2 (11)	*p* > 0.999
Mental disorder	10 (10)	9 (11)	1 (5)	*p* = 0.682
Misc.	9 (9)	8 (10)	1 (5)	*p* > 0.999
Injury characteristics				
Months since injury, mean (SD)	7.42 (3.89)	6.95 (3.71)	9.44 (4.07)	***p* = 0.010**
GCS, *n* (%)				
15	92 (92)	74 (96)	18 (95)	*p* > 0.999
14	4 (4)	3 (4)	1 (5)	
PTA, *n* (%)				
No	67 (67)	55 (68)	12 (67)	*p* > 0.999
Yes	30 (30)	24 (30)	6 (33)	
LOC, *n* (%)				
No	74 (74)	61 (75)	13 (72)	*p* = 0.785
Yes	25 (25)	20 (25)	5 (28)	
Imaging (CT/MR), n (%)				
No	15	12 (15)	3 (16)	*p* = 0.915
Yes	85	69 (85)	16 (84)	
Complicated mTBI, *n* (%)				
No	96 (96)	78 (97.5)	18 (94.7)	*p* = 0.476
Yes	3 (3)	2 (2.5)	1 (5.3)	
Injury mechanism, *n* (%)				
Fall	31 (31)	27(33.3%)	4 (21%)	*p* = 0.225
Traffic or bike accident	18 (18)	17 (21%)	1 (5%)	
Sports-related injury	21 (21)	14 (17.3%)	7 (37%)	
Violence	4 (4)	4 (5%)	0 (0%)	
Misc.	26 (26)	19 (23.5%)	7 (37%)	

BMI: body mass index (kg/m^2^). Blood pressure groups retrieved from European Society of Cardiology (19). GCS: Glasgow Coma Scale; PTA: post traumatic amnesia; LOC: loss of consciousness; mTBI: mild traumatic brain injury. Bold indicates statistical significance, *p*<0.05.

The mean sum score of RPQ was 28.7 (SD: 10.2) corresponding to moderate to severe PCS. The median number of PCS items scored as moderate or severe was 6 (p25: 3, p75: 8). HRQOL was low with a QOLIBRI-OS mean score of 39.1 (SD: 17.2). Depression scores, as measured with PHQ-9, corresponded to symptoms of moderate depression with a mean score of 10.0 (SD: 4.2). Anxiety symptoms, as measured with GAD-7, were mild with a mean score of 6.4 (SD: 3.9). Fatigue severity was high with a mean score of 5.1 (SD: 1.1) For detailed patient symptoms and functioning see Table II.

**Table II:** Comparison between exercise intolerant and exercise tolerant groups on patient-reported outcome measures and Buffalo Concussion Treadmill Test

Item	Overall	Exercise intolerant *n* = 81	Exercise tolerant *n* = 19	*P*-value
Patient-reported outcome measures				
RPQ sum score, mean (SD)	28.66 (10.16)	29.7 (9.23)	24.4(12.9)	***p* = 0.043**
QOLIBRI-OS, mean (SD)	39.1 (17.2)	36.8 (15.4)	46.71 (23.8)	***p* = 0.027**
PHQ-9, mean (SD)	10.03 (4.21)	10.5 (4.1)	8.11 (4.2)	***p* = 0.026**
GAD-7, mean (SD)	6.36 (3.94)	6.71 (3.9)	4.89 (3.8)	*p* = 0.061
FSS, average, mean (SD)	5.13 (1.13)	5.14 (1.1)	5.5 (1.5)	*p* = 0.757
IPAQ: MET-min/week, median (IQR)	1631(790–2,750)	1650 (798–2,565)	1,620 (830–3,426)	*p* = 0.607
IPAQ sit hours, median (IQR)	8.03 (5.25–10)	8 (5–10)	8 (7–8.75)	*p* = 0.900
Buffalo Concussion Treadmill Test				
Symptom threshold, mean (SD)		71.1 (10.7)	90 (2)	***p* < 0.001**
BCTT HR_0_ (bpm), median (IQR)		77 (69–90)	81 (73–90)	*p* = 0.335
Borg RPE at stop, mean (SD)		12.9 (2.2)	16.2 (1.3)	***p* < 0.001**
Minutes-to-stop, mean (SD)		8.2 (4.4)	15.4 (4.8)	***p* < 0.001**

RPQ: Rivermead Post-Concussion Symptoms Questionnaire; PHQ-9: Patient Health Questionnaire 9-items; GAD-7: Generalized Anxiety Disorder 7; FSS: Fatigue Severity Scale; IPAQ: International Physical Activity Questionnaire (activity level categories of MET minutes per week, and hours spent sitting), Symptom threshold: HR at stop adjusted by age; Borg RPE: Borg Rating of Perceived Exertion 6–20; IQR: interquartile range. Bold indicates statistical significance, *p*<0.05.

### Exploring exercise intolerance assessed with Buffalo Concussion Treadmill Test

**Fig. 1:**
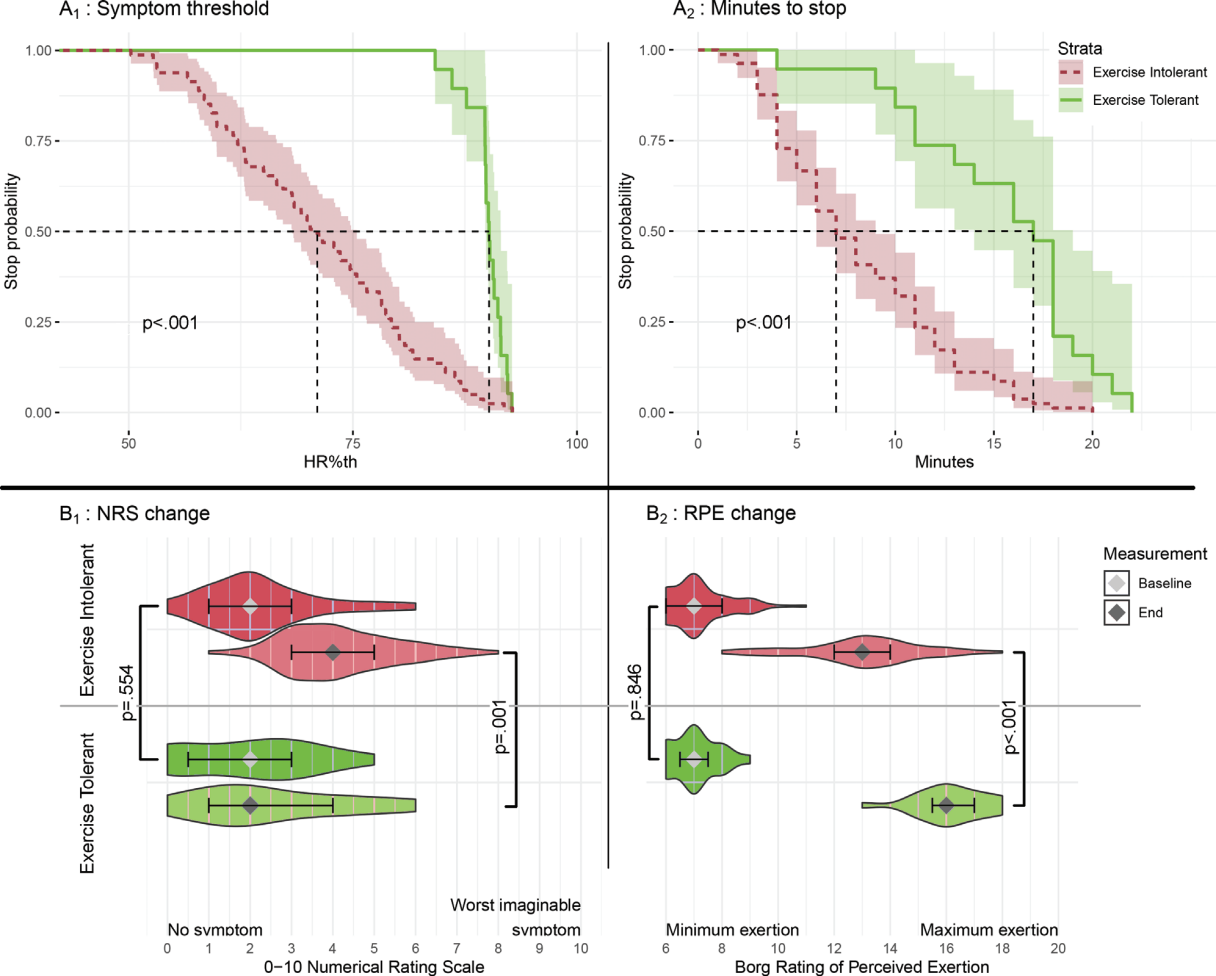
Buffalo Concussion Treadmill Test: symptom threshold and minutes-to-stop. A_1_: Kaplan–Meier plot of symptom threshold during BCTT, compared per exercise-intolerant and exercise-tolerant group. Statistics A_2_: Kaplan–Meier plot of minutes-to-stop of BCTT, compared per exercise-intolerant and exercise-tolerant group. Faded area = 95% CI. B figures: illustrates the difference in NRS measure and Borg RPE measures at baseline and end of BCTT, compared per exercise-intolerant and exercise-tolerant group. B_1&2_: Violin plots: Stars = median, error bar = interquartile range (IQR). Test statistics: B_1_: NRS 0–10: baseline, *t* = –0.602, *p* = 0.554; end: *t* = 3.69, *p* = 0.001. B_2_: Borg RPE 6–20, baseline: *t* = 0.196, *p* = 0.846; At stop, *t* = 8.48, *p* < 0.001.

On the BCTT, 81 patients were EI and 19 ET. Symptom threshold, time-to-stop, NRS 0–10, and Borg RPE recordings from BCTT are shown in Fig. 1 and reported in Table II. At test stop, the EI group had significantly lower HR (symptom threshold for EI), Borg RPE, and time-to-stop (all *p* < 0.001) and reported significantly more new exacerbating symptoms (median 2 [p25: 1, p75: 2] vs median 1 [p25: 1, p75: 1], W = 1178, *p* < 0.001). In the ET group, 3 reported symptom improvement during the test, 7 had no change, and 9 patients reported a 1–2-point increase on the NRS 0–10. The most common exacerbating symptoms in the EI group were headache, dizziness, and vision symptoms. No adverse events were registered in relation to the BCTT.

The EI group was younger by a mean of 6 years (*p* = 0.02), had a lower proportion of patients with hypertension (18.5% vs 42%) and more often normal blood pressure (42% vs 21%, *p* = 0.045) at test start. They had 2.4 months shorter time-since-injury (*p* = 0.01) (see Table I).

The EI group reported a significantly higher symptom burden with 5.3 points higher sum score on the RPQ. The EI group also reported a significantly poorer HRQOL with a 9.9 lower sum score on the QOLIBRI-OS and had significantly more psychological distress on the PHQ-9 (*p* = 0.026). Both groups reported similar and high fatigue on the FSS-9 (*p* = 0.757), and comparable physical activity levels (*p* = 0.900) (see Table II).

### Association between BCTT results and persistent PCS burden

In the logistic regression model assessing the association between the positive and negative BCTT (EI/ET group as the dependent variable) and RPQ sum score (*n* = 100), we found a significant association between testing positive for EI and a higher symptom burden measured with the RPQ sum score. Each 1-point increase on the RPQ was associated with 6.6% higher odds of testing positive for EI on the BCTT (OR: 1.066 [95% CI: 1.01–1.13], *p* = 0.023). Model fit (Hosmer–Lemeshow) was satisfactory (*p* = 0.164).

### Factors associated with BCTT symptom threshold in patients with exercise intolerance

The multiple linear regression model presented in Table III, exploring factors associated with the BCTT symptom threshold within the EI group (*n* = 81), explained 21% of the variation in the symptom threshold (adj. R^2^: 0.21, *p* < 0.001). The VIF scores between RPQ and PHQ-9 showed mild overlap and were deemed as generally acceptable < 5. The following variables contributed significantly to the model: BCTT HR_0_ (*B*: 0.34, 95% CI: 0.17 to 0.50, *p* < 0.001) and GAD-7 (*B*: –0.86, 95% CI: –1.6 to –0.12, *p* < 0.023) showing that a higher BCTT HR_0_was associated with a higher symptom threshold and more anxiety was associated with a lower symptom threshold.

**Table III:** Buffalo Concussion Treadmill Test: symptom threshold in the exercise-intolerant group (multiple linear regression, *n* = 81)

Item	Full model				
	*β*	95% CI		*p*-value	VIF
Intercept	49.16	29.39	68.93	**< 0.001**	
Sex					
– Female	Ref.	–	–	0.448	1.21
– Male	–1.80	–6.51	2.90		
Months since injury	–0.19	–0.84	0.46	0.560	1.30
BCTT: HR_0_	0.34	0.17	0.50	**< 0.001**	1.09
Rivermead Post Concussion Symptom Questionnaire, sum score	–0.01	–0.34	0.33	0.969	2.11
Patient Health Questionnaire-9	0.55	–0.24	1.35	0.170	2.37
Generalized Anxiety Disorder-7	–0.86	–1.61	–0.12	**0.023**	1.87
Fatigue Severity Scale	–0.43	–2.78	1.92	0.717	1.36
International Physical Activity Questionnaire: MET-h	–0.0001	–0.002	0.001	0.870	1.05
	AIC: 605.1, multiple R^2^: 0.29, adjusted R^2^: 0.21				

Unstandardized regression coefficients (β) symptom threshold: BCTT: HR at stop, adjusted by age. BCTT HR_0:_ heart rate prior to start of BCTT; PHQ-9: Patient Health Questionnaire 9-items; GAD-7: Generalized Anxiety Disorder 7; FSS: Fatigue Severity Scale; IPAQ: International Physical Activity Questionnaire (MET-hours); VIF: variance inflation factors. Bold indicates statistical significance, *p*<0.05.

## DISCUSSION

In this cross-sectional study, we assessed EI and associated factors in patients with mTBI. We found that 81% of patients who reported symptom exacerbation with physical activity tested positive for EI on the BCTT. The presence of EI was associated with higher PCS burden. Additionally, a higher BCTT HR_0_ was associated with a higher symptom threshold on the BCTT, while greater anxiety symptoms were linked to a lower symptom threshold.

### Exploration of exercise intolerance

Our findings support that EI is a distinct condition within PCS (1). Some 81% of the included patients were confirmed as exercise intolerant by the BCTT and this supports the understanding that EI, previously observed in the subacute phase among young athletes (1, 9), also persists in adult patients with PPCS several months post-injury (7).

The moderate to severe symptom burden found in the current study indicates that EI is part of a more severe and persisting symptom complex post-injury. The fact that the EI group scored higher on the RPQ than the ET group supports this. Furthermore, compared with a CENTER-TBI study (31), the EI group scored higher on the RPQ and had considerably lower HRQOL. The low HRQOL was also found in a study from Canada testing EI patients (7), suggesting that EI patients experience lower HRQOL than the general population with persistent PCS.

When comparing the EI and ET groups, we found a higher score for depression and anxiety in the EI group. This is supported by other studies (3, 32, 33), which found associations between poorer BCTT performance and more severe symptoms of anxiety and depression, indicating increased psychological distress with EI. Importantly, 1 study found that after sustaining a mTBI and having EI concurrent anxiety symptoms were associated with PCS, while pre-morbid anxiety was not (33). Other studies have found a complex interplay linking depression and anxiety to a pre-morbid factor for psychological distress, in addition to being specific symptoms within PCS, and a consequence of PCS (3, 11, 34). This further supports that EI is part of a complex phenomenon of persistent PCS, in which bidirectional relationships should be disentangled to facilitate clinical decision-making in relation to therapeutic targets.

The current study found shorter time-since-injury in the EI group, suggesting an improvement in exercise intolerance over time. This aligns with cohort studies in both Europe (6) and the United States (35) that have found a decrease in PCS over time. Hence, it is reasonable to assume that EI as part of a persisting symptom complex also decreases with time. The impact of time-since-injury is complex and must be seen in relation to other factors involved in maintaining the symptoms, such as advice given to patients earlier in the trajectory, chronification development, psychological distress, and types of symptoms experienced (36).

The proportion of 81% of patients with EI was higher in the current study compared with other studies (3, 7, 11, 12, 32, 33), which ranged between 30% and 58%. There might be several reasons for this difference. First, our study included only patients with persistent PCS after mTBI referred to a TBI clinic in specialized healthcare and with self-reported exacerbation of symptoms, representing a more selected population. Second, methodological differences in the BCTT protocol might affect the cut-off of what constitutes a positive test. Hence the proportion of patients with EI included in the different studies may differ due to variations in stop criteria, such as different HRmax cut-offs (3), Borg RPE cut-offs (12), minutes to stop (11), and use of the bike version of the test (32, 33).

Our study showed that the EI group reported more exacerbating PCS. Also, for HR, ratings of perceived exertion on Borg RPE, and time to stop, the EI group performed more poorly than the ET group. -However, that is the exact purpose of the BCTT. Hence, BCTT effectively separated the patients into EI and ET groups. This is in line with findings from several studies of mTBI populations and EI in different age spans and phases post-injury (1–3, 11, 12, 32, 33, 37, 38), suggesting that a symptom-limited incremental test is necessary for proper diagnosis and management of exercise intolerance. Furthermore, it confirms that symptom reports alone are not sufficient to detect EI, and that incremental tests utilizing symptom thresholds can provide important information about EI that might otherwise go undetected (15, 16). Importantly, the BCTT provides a symptom threshold that can be regarded as the severity of the EI which can guide exercise intensity prescription (7, 38).

The patients with EI walked a mean of 8.2 (SD: 4.4) min on the treadmill, which is in accordance with other studies reporting means from 6.6 to 12 min (3, 12, 32). Furthermore, consistent with previous studies (11, 12, 32), the range of minutes-to-stop in the current study was between 1 and 21 min. This highlights individual variations within the EI group. This large variation makes minutes-to-stop on the BCTT a problematic outcome for comparisons in the adult general mTBI population, thus supporting symptom threshold as a more suitable outcome for the BCTT.

### Association between BCTT results (EI/ET) and persistent PCS burden

As previously discussed, the BCTT effectively grouped patients into ET or EI. The logistic regression assessing association between positive and negative BCTT with the RPQ sum score, adjusted for age and sex, showed that higher RPQ sum-scores increased the odds of testing positive on the BCTT by 6.6%. This aligns with other studies linking higher symptom burden to poorer exercise test results and increased likelihood of EI (2, 11, 33). Symptom exacerbation of PCS-related symptoms due to walking on a treadmill are necessary for a positive BCTT. It has been suggested that symptom burden and symptoms reported in relation to activities are separate entities (39) that requires different approaches. Our result shows that exercise intolerance is associated with a higher symptom burden, thus exercise intolerance should be considered in patients who report more PCS.

### Factors associated with the BCTT symptom threshold in patients with exercise intolerance

Symptom thresholds in the EI group ranged from 50% to 90% of estimated HRmax, which indicates symptoms exacerbating at activity levels ranging from walking to running. Our finding of mean symptom threshold at 71.1% (SD: 10.7) of HRmax aligns with comparable studies that reported mean symptom thresholds between 71.6% and 75.6% (7, 12, 32).

Within the EI patients, a higher BCTT HR_0_, which can be thought of as reflecting a state of activation of the sympathetic autonomic nervous system (15), was positively associated with a higher symptom threshold, showing a better test result on the BCTT. In our model, a 10-bpm higher BCTT HR0 corresponded to a 3.4 percentage-point higher BCTT symptom threshold. This finding is supported by a recently published study comparing patients with persistent PCS with healthy controls finding evidence for autonomic cardiovascular dysfunction in patients with persistent PCS (40). Other studies have found conflicting or unclear results (12, 15, 33). In the current study each one-unit higher score for anxiety on the GAD-7 was associated with a lower symptom threshold, indicating poorer performance on the BCTT. This result confirms the role of anxiety in EI in this group of patients. Interestingly, although a higher RPQ sum score was associated with having EI when comparing the groups, it was not associated with the symptom threshold within the EI group. When comparing the groups, it was not associated with the symptom threshold within the EI group.

Only 21% of the variance in BCTT symptom threshold was explained by the regression model. Other factors that may contribute to the unexplained variance are physiological factors including measures of autonomic dysfunction and central sensitization. Furthermore, the inclusion of more specific measures of fear-avoidance and fear of movement might have explained a greater proportion of the variance in BCTT symptom threshold.

Together, our findings support that the BCTT symptom threshold can be used as an indicator of the severity of EI. However, the association between BCTT symptom threshold and activity limitations in real life should be explored in future studies. Findings in studies on adolescents imply that a lower symptom threshold is associated with worse clinical outcome (41). However, replications of such findings are needed in adults. The natural course of EI and its symptom threshold after injury are unknown, although a recent RCT reports an improvement during 6 weeks’ intervention in both intervention and control group (7).

### Fatigue and physical activity

Contrary to our hypothesis, although both groups reported above the cut-off for high fatigue, there were no significant differences between the EI and ET groups. Severity of fatigue was not a significant variable in the regression model. While other studies have linked fatigue to EI (3, 12, 32, 33), our findings did not support this association.

Deconditioning has been theorized as a cause of the autonomic dysfunction involved in EI (15, 17). We hypothesized that higher levels of post-injury physical activity would indicate less EI through mitigation of deconditioning, but we did not find evidence for this. This is supported by De Groot et al. (11), who found no correlation between physical activity level and BCTT duration. Further, a cohort study by Mercier et al. (42) found no correlation between RPQ sum scores and physical activity levels, while Galea et al. (12) found higher physical activity levels in EI patients compared with healthy controls. Future high-quality studies are needed to clarify these relationships. Until then, caution is called for in attributing EI to deconditioning, as evidence remains scarce.

This cross-sectional study has limitations, including the inability to infer causal relationships, particularly for time-dependent factors that may be interrelated. We recruited a selected group of adult patients with persistent PCS who were motivated to participate in an RCT involving aerobic exercise. Patients referred to the TBI outpatient clinic in specialized healthcare represent a patient group with more severe PCS and should not be generalized to the larger unselected mTBI population. We did not include patients aged 60 or more or adolescents below the age of 18, further limiting the generalizability of this study. The sample size of 81 patients limited the number of independent variables in the regression model; however, we adhered to the rule-of-thumb of 10 patients per variable. In conclusion, EI was confirmed by the BCTT in over 80% of adult patients with persistent PCS and self-reported symptom exacerbation during physical activity. The likelihood of EI increased with higher PCS burden. The model exploring symptom threshold resulted in largely unexplained variance; however, greater anxiety was associated with a lower symptom threshold and higher BCTT HR0 higher symptom threshold. More research is needed to understand which factors contribute to symptom threshold, and the clinical implications in patients with persisting PCS. These findings suggest that patients with persistent PCS and exacerbation during physical activity should be assessed for EI to support individualized rehabilitation strategies.
